# Cutaneous Plasmacytosis: A Rare Dermatological Condition Mimicking Systemic Plasma Cell Disorders

**DOI:** 10.7759/cureus.75948

**Published:** 2024-12-18

**Authors:** Ian Depew, William T Snider, Shane Cook, Olivia Lim

**Affiliations:** 1 Dermatology, Marshall University Joan C. Edwards School of Medicine, Huntington, USA

**Keywords:** cutaneous plasmacytosis, igg4-related disease, polyclonal plasma cells, systemic evaluation, topical corticosteroids

## Abstract

Cutaneous plasmacytosis (CP) is a rare condition characterized by benign proliferation of mature plasma cells in the skin. It presents as reddish-brown macules, papules, or plaques, typically located on the neck, face, and trunk. The etiology remains unknown, though it is believed to be reactive rather than malignant. CP can occasionally become systemic, affecting two or more extracutaneous organs. This report describes a 54-year-old Caucasian male patient with erythematous, scaly plaques on his lower back and arms. Histopathology revealed a dense polyclonal plasma cell infiltrate with a predominance of IgG4+ cells. The patient was treated with topical corticosteroids and referred for further systemic workup. This case emphasizes the importance of distinguishing CP from systemic plasmacytosis, which can mimic multiple myeloma, plasmacytoma, or Waldenstrom’s macroglobulinemia, each requiring distinct management approaches.

## Introduction

Cutaneous plasmacytosis (CP) is an uncommon dermatological condition marked by benign plasma cell infiltrations in the skin. It typically manifests as multiple reddish-brown nodules or plaques, most commonly on the trunk and face [1]. While these lesions are usually asymptomatic, some may cause pruritus or discomfort. CP is associated with polyclonal hypergammaglobulinemia and superficial lymphadenopathy but lacks systemic organ involvement, distinguishing it from systemic plasmacytosis [2].

Histopathological analysis reveals polyclonal plasma cells, without evidence of monoclonality or malignancy, which is crucial for diagnosis [3]. Systemic plasmacytosis involves polyclonal plasma cell infiltrates across multiple organs, including lymph nodes, bone marrow, and lungs [4]. Differential diagnoses for CP include plasmacytoma, multiple myeloma, primary cutaneous B-cell lymphoma, T-cell lymphoma, leprosy, secondary syphilis, and leishmaniasis [5]. Proper patient history, symptom evaluation, and biopsy are vital for differentiating these conditions, as malignant plasma cell disorders often exhibit monoclonal proliferation, lytic bone lesions, and rapid progression, requiring aggressive treatment [6].

This report presents a case of CP in a 54-year-old male patient, detailing the diagnostic process and the importance of ruling out systemic involvement for accurate management and avoiding misdiagnosis of plasma cell dyscrasias.

## Case presentation

**Figure 1:**
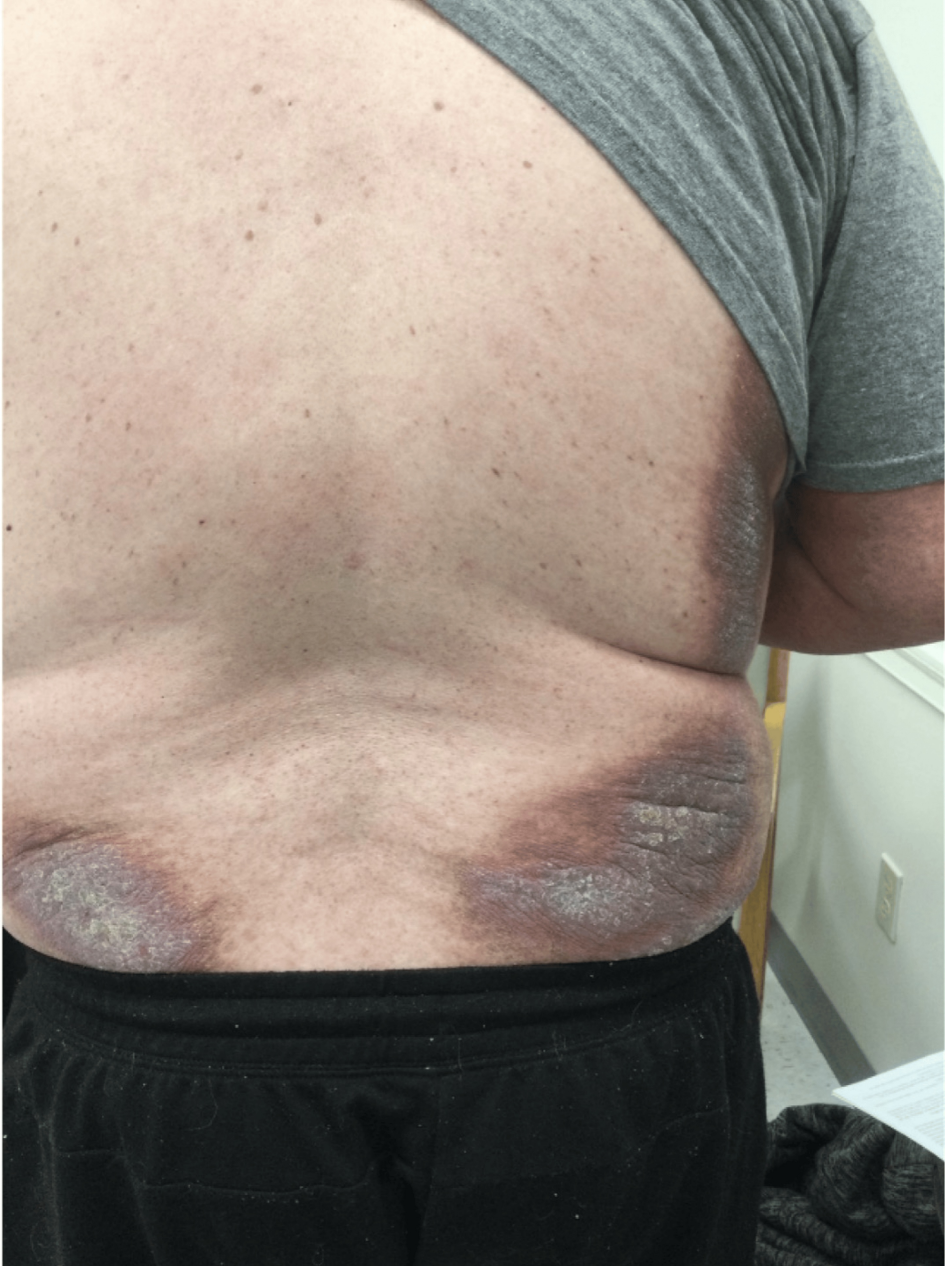
Initial presentation of the patient's rash, characterized by erythematous, scaly plaques on the bilateral lower back.

**Figure 2:**
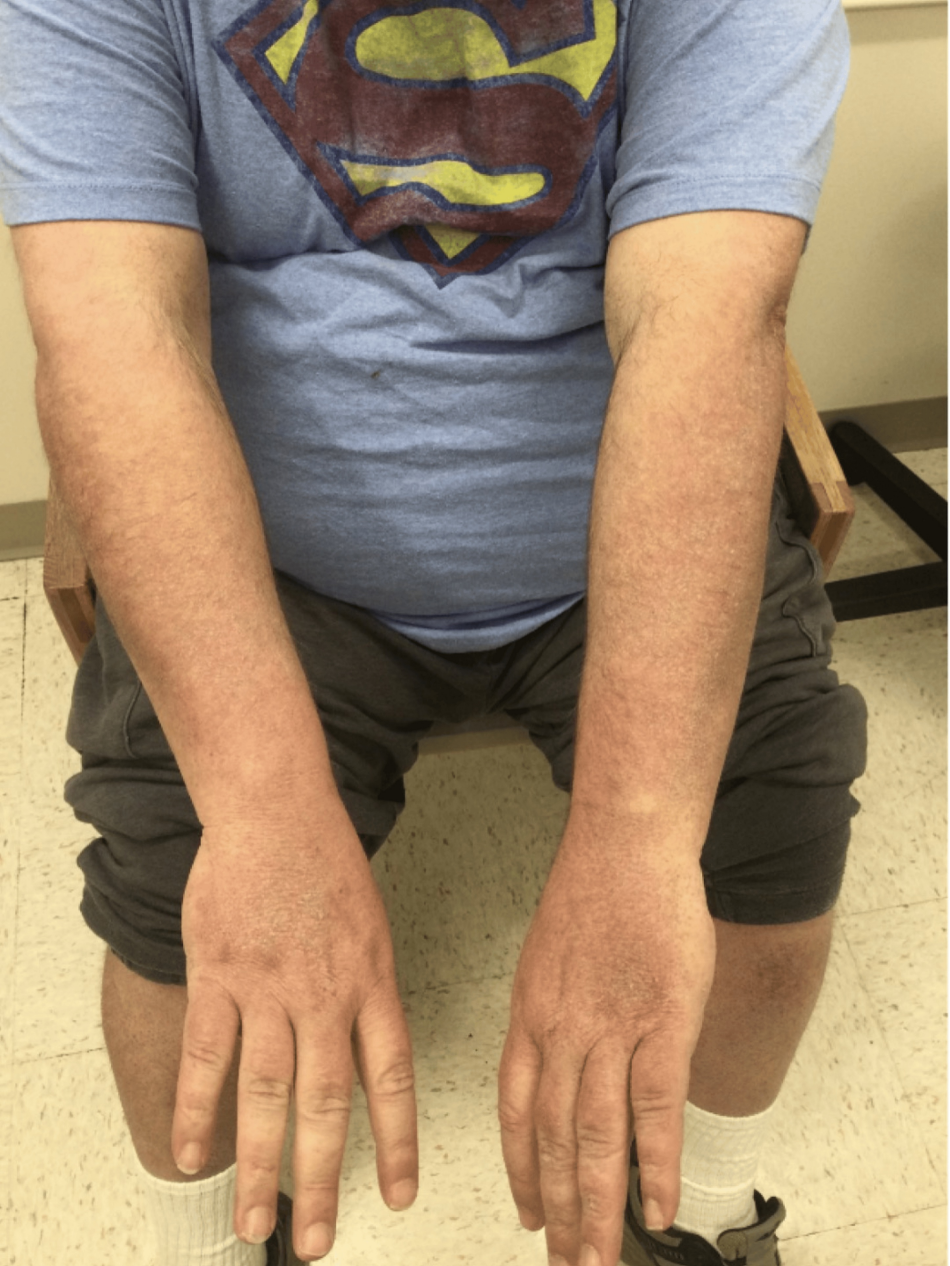
Macular hyperpigmentation along the dorsal forearms. Active macular erythema is evident on the dorsal forearms, consistent with post-inflammatory changes and residual disease activity.

A 54-year-old Caucasian male patient with a medical history of chronic obstructive pulmonary disease (COPD) presented with a several-month history of erythematous, scaly plaques on his lower back and macular erythema on his arms (Figures 1, 2). Initial shave biopsies revealed a dense, polytypic plasma cell infiltrate with minor populations of lymphocytes, histiocytes, and eosinophils, confirmed as polyclonal via immunohistochemistry. Follow-up punch biopsies demonstrated a dense lymphoplasmacytic infiltration with prominent IgG4-positive cells, suggesting cutaneous plasmacytosis (CP) or IgG4-related disease. Despite treatment with topical mometasone and intralesional Kenalog, the patient reported inconsistent application and only mild improvement. He also disclosed a flea and bed bug infestation at home, linking some lesions to insect bites. Further systemic evaluation, including serum protein electrophoresis (SPEP), imaging studies, and a bone marrow biopsy, ruled out monoclonal plasma cell disorders, confirming the diagnosis of cutaneous plasmacytosis with elevated IgG4 levels.

**Figure 3:**
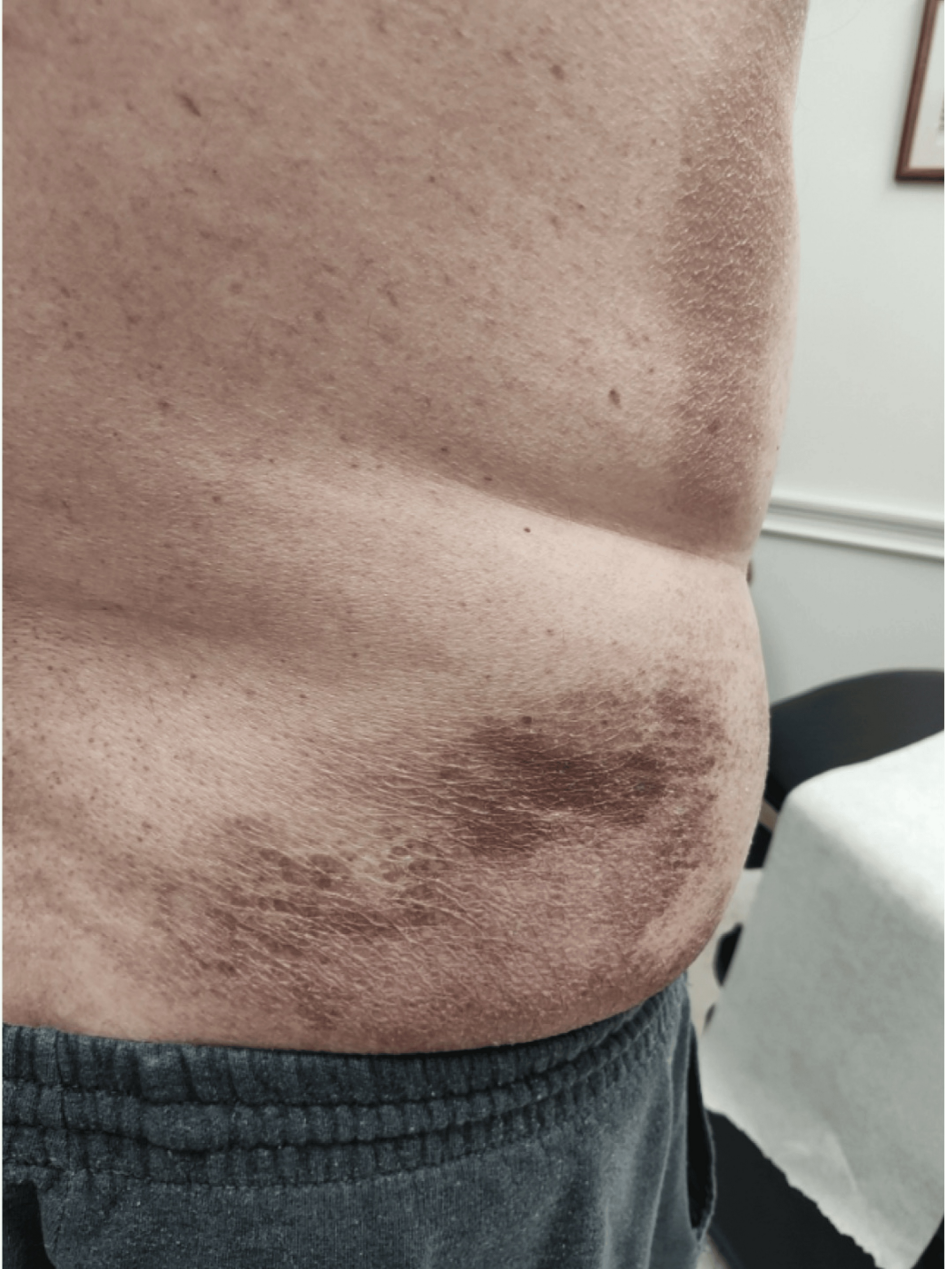
Follow-up clinical images demonstrating significant improvement in pruritus and overall lesion clearance with notable resolution of plaques on the back.

**Figure 4:**
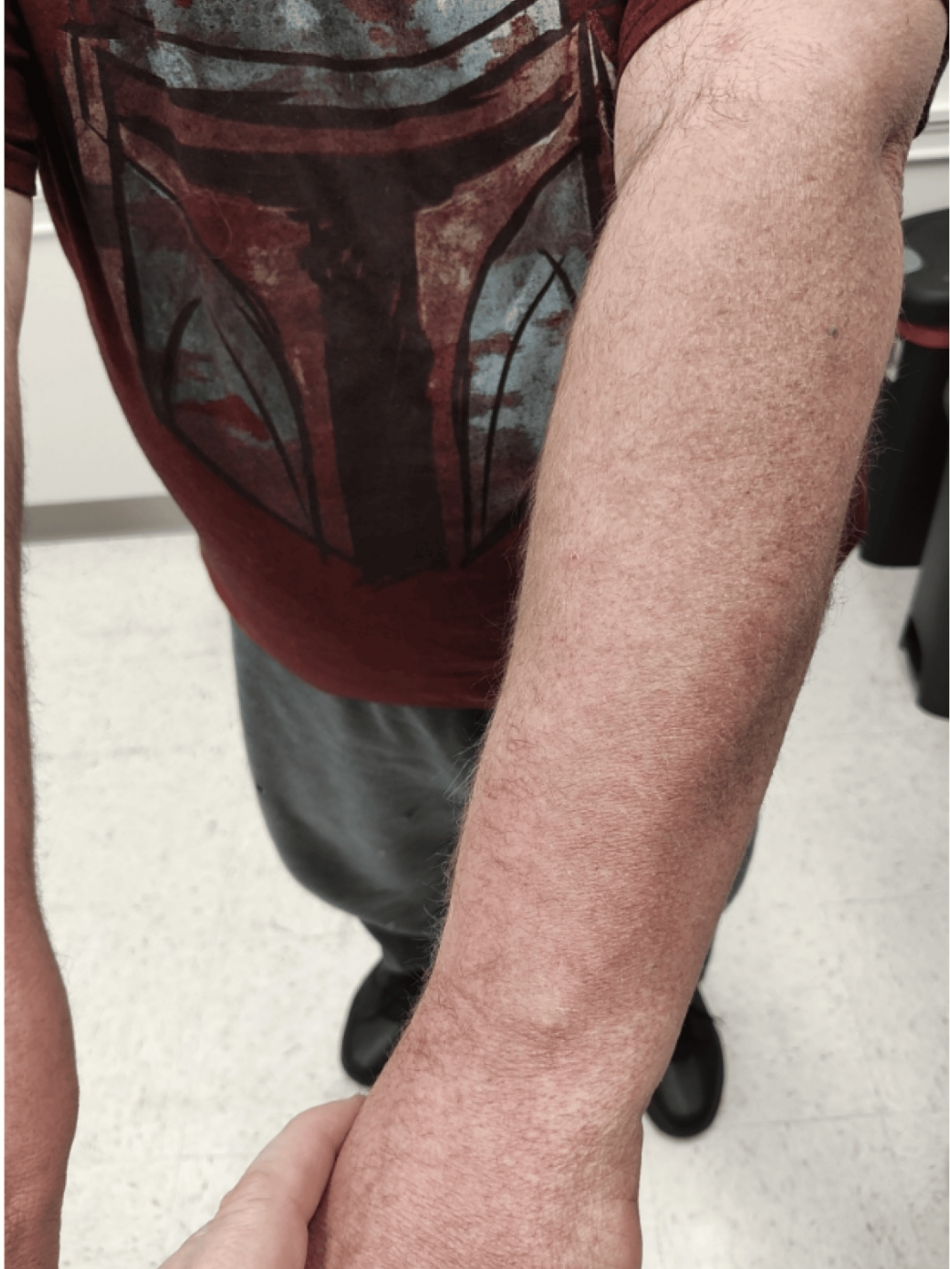
Follow-up clinical images demonstrating significant improvement in pruritus and overall lesion clearance. Mild increased redness persists on the dorsal forearms.

Over the subsequent months, the patient received intralesional steroid injections, oral steroids, and topical treatments, showing gradual improvement in his skin lesions. Imaging studies and lymph node biopsies identified mild adenopathy and IgG4-related lymphadenopathy without systemic organ involvement. Follow-up visits documented significant clearance of plaques on his back, though recurrent erythema on his dorsal forearms persisted (Figures 3, 4). Rheumatologic evaluation confirmed underlying IgG4-related disease with primary skin involvement but no need for DMARD (Disease-Modifying Antirheumatic Drug) therapy. Continued treatment with intralesional steroids and topical therapies resulted in marked symptom improvement, including reduced pruritus and lesion resolution, underscoring the chronic but manageable nature of this condition.

## Discussion

This case presents a classic CP profile, with erythematous plaques and histological findings of polytypic plasma cell infiltrates, predominantly IgG4+ [7]. Diagnosing CP is challenging due to its similarity to systemic plasmacytosis and monoclonal plasma cell disorders like multiple myeloma [5]. CP can mimic conditions such as pseudolymphomas or cutaneous T-cell lymphoma, making histopathological evaluation crucial for accurate diagnosis.

Systemic plasmacytosis typically presents with symptoms such as lymphadenopathy, hepatosplenomegaly, fever, fatigue, and weight loss, while multiple myeloma often involves monoclonal gammopathy, lytic bone lesions, and secondary amyloidosis. Immunohistochemistry helps differentiate CP, revealing CD79a positivity and a kappa:lambda ratio of 2:1 or 3:1, indicating polyclonality [2,7]. The significant IgG4+ cell predominance further supported the diagnosis.

Histopathological evaluation typically shows a dense plasma cell infiltrate in the dermis with perivascular accentuation, fibrosis, and varying lymphocytes and histiocytes [8]. While IgG4+ cells indicate overlap with IgG4-related disease, CP is limited to the skin and lacks the systemic fibrotic changes seen in IgG4-related disease. In this case, clinical, histological, and immunohistochemical findings confirmed CP and highlighted its benign nature when systemic involvement is excluded.

A systemic workup, including SPEP, imaging, and bone marrow biopsy, is essential to rule out extracutaneous involvement. Systemic plasmacytosis and monoclonal plasma cell disorders can present similarly to CP in early stages [9].

Treatment for CP is primarily conservative, focusing on cutaneous lesions. Topical corticosteroids are effective first-line treatments, and intralesional triamcinolone or low-dose thalidomide can be used for refractory lesions [10,11]. Systemic corticosteroids or immunosuppressants may be considered in extensive cases. Narrowband UVB (Ultraviolet B) or PUVA (Psoralen Ultraviolet A) therapy may also aid in reducing inflammation [12].

In this case, topical and intralesional corticosteroids effectively managed the patient's lesions, emphasizing CP's manageable nature when systemic involvement is absent. Systemic plasmacytosis, however, remains a concern due to its chronic course and potential organ dysfunction, underscoring the need for vigilance and systemic evaluation.

## Conclusions

Cutaneous plasmacytosis is a rare, benign condition characterized by polyclonal plasma cell infiltrates in the skin, with a clinical presentation resembling other plasma cell disorders. This case report emphasizes the importance of distinguishing CP from systemic involvement, which can mimic malignancies like multiple myeloma or plasmacytoma. Through a comprehensive diagnostic workup, including clinical evaluation, histopathology, and immunohistochemistry, accurate diagnosis can be achieved, ensuring appropriate management. In this patient, localized treatments such as topical corticosteroids and intralesional injections provided effective symptom control, highlighting CP's manageable nature when systemic disease is excluded. While CP is typically a self-limited disorder, vigilance for possible systemic involvement remains crucial, underscoring the need for continued research into its pathophysiology and treatment strategies.
